# Ethical considerations for HIV cure-related research at the end of life

**DOI:** 10.1186/s12910-018-0321-2

**Published:** 2018-10-20

**Authors:** Karine Dubé, Sara Gianella, Susan Concha-Garcia, Susan J Little, Andy Kaytes, Jeff Taylor, Kushagra Mathur, Sogol Javadi, Anshula Nathan, Hursch Patel, Stuart Luter, Sean Philpott-Jones, Brandon Brown, Davey Smith

**Affiliations:** 1 Gillings School of Global Public Health, University of North Carolina, 4108 McGavran-Greenberg Hall, Chapel Hill, North Carolina USA; 2 Division of Infectious Diseases and Global Public Health, University of California San Diego, Stein Clinical Research Building, La Jolla, California USA; 3 AntiViral Research Center (AVRC), University of California San Diego, 220 Dickinson Street, Suite A, San Diego, California USA; 4 AVRC Community Advisory Board, University of California San Diego, 220 Dickinson Street, Suite A, San Diego, California USA; 5 HIV and Aging Research Project – Palm Springs (HARP-PS), 1775 East Palm Canyon Drive, Suite 110-349, Palm Springs, California USA; 6 Department of Bioethics, Clarkson University, 80 Nott Terrace, Schenectady, New York USA; 7 Center for Healthy Communities, Department of Social Medicine, Population, and Public Health, University of California Riverside School of Medicine, 3333 14th Street, Riverside, California USA

**Keywords:** HIV cure research, End-of-life (EOL), Last gift, Rapid research autopsy, Altruism, Ethical considerations

## Abstract

**Background:**

The U.S. National Institute of Allergies and Infectious Diseases (NIAID) and the National Institute of Mental Health (NIMH) have a new research priority: inclusion of terminally ill persons living with HIV (PLWHIV) in HIV cure-related research. For example, the Last Gift is a clinical research study at the University of California San Diego (UCSD) for PLWHIV who have a terminal illness, with a prognosis of less than 6 months.

**Discussion:**

As end-of-life (EOL) HIV cure research is relatively new, the scientific community has a timely opportunity to examine the related ethical challenges. Following an extensive review of the EOL and HIV cure research ethics literature, combined with deliberation from various stakeholders (biomedical researchers, PLWHIV, bioethicists, and socio-behavioral scientists) and our experience with the Last Gift study to date, we outline considerations to ensure that such research with terminally ill PLWHIV remains ethical, focusing on five topics: 1) protecting autonomy through informed consent, 2) avoiding exploitation and fostering altruism, 3) maintaining a favorable benefits/risks balance, 4) safeguarding against vulnerability through patient-participant centeredness, and 5) ensuring the acceptance of next-of-kin/loved ones and community stakeholders.

**Conclusion:**

EOL HIV cure-related research can be performed ethically and effectively by anticipating key issues that may arise. While not unique to the fields of EOL or HIV cure-related research, the considerations highlighted can help us support a new research approach. We must honor the lives of PLWHIV whose involvement in research can provide the knowledge needed to achieve the dream of making HIV infection curable.

## Background

Due to the development of highly effective antiretroviral therapy (ART), persons living with HIV (PLWHIV) are living longer [1–3]. In the United States, half of PLWHIV will be 50 years or older by the year 2020 [3]. Considered an almost certain death sentence in the 1980s, HIV/AIDS has now become a manageable chronic condition. PLWHIV increasingly die of causes mirroring those in the general population [4]. Despite this, HIV persists throughout the body [5] and a global effort is underway to find a cure for the infection [6].

The U.S. Food and Drug Administration (FDA) defines HIV cure research as “any investigation that evaluates: 1) a therapeutic intervention or approach that controls or eliminates HIV infection to the point that no further medical interventions are needed to maintain health; and 2) preliminary scientific concepts that might ultimately lead to such a therapeutic intervention [7].” HIV cure or remission research includes both observational assessments, such as the measurement and characterization of HIV reservoirs, and interventional approaches, including early ART, latency-reversing agents, immune-based strategies, gene editing or modification, stem cell transplantation, or combination approaches [6]. While most HIV cure-related studies occur in ‘otherwise healthy volunteers’ [8], a new research priority for the U.S. National Institute of Allergies and Infectious Diseases (NIAID) and the National Institute of Mental Health (NIMH) involves the inclusion of PLWHIV who are terminally ill [9, 10]. End-of-life (EOL) HIV cure research is currently limited to observational assessments at one clinical research site in the United States: The University of California, San Diego (UCSD). The purpose of this research is to investigate HIV reservoirs and does not involve palliative care research. EOL HIV cure-related research may soon be expanded to include additional clinical research sites throughout the U.S. and to involve interventional HIV cure-related approaches, such as broadly neutralizing antibodies and other interventions with a favorable benefit/risk assessment. This research does not benefit participants physically, but seeks to produce generalizable scientific knowledge towards an HIV cure or virologic suppression off therapy.

While clinical research with participants at the end of life is not a new phenomenon [11, 12], the utilization of this model in the setting of HIV cure research is novel. Since EOL HIV cure research is relatively new, the scientific community has a timely opportunity to examine ethical challenges associated with this type of research. Our paper is rooted in the ethics of conducting clinical research at the EOL [11, 12], and builds on an emerging HIV cure research ethics literature [13–15]. Following an extensive review of the EOL and HIV cure research ethics literature, combined with deliberation from various stakeholders (biomedical researchers, PLWHIV, bioethicists, socio-behavioral scientists and Community Advisory Board members of the AntiViral Research Center at UCSD, the Palm Springs HIV and Aging Research Project and the Palm Springs Positive Life Program), as well as our experience in the Last Gift study to date, we describe possible ethical considerations for the design, conduct, review and evaluation of HIV cure-related research with terminally ill PLWHIV. After describing EOL HIV cure-related research at UCSD [10] and identifying ethical issues for clinical research at the EOL, we review topics relevant to HIV cure-related research with terminally ill PLWHIV.

We outline considerations to ensure that such research remains ethical, focusing on five domains: 1) protecting autonomy through informed consent, 2) avoiding exploitation and fostering altruism, 3) maintaining a favorable benefits/risks balance, 4) safeguarding against vulnerability through patient-participant centeredness, and 5) ensuring the acceptance of next-of-kin/loved ones and community stakeholders. We conclude by summarizing ethical considerations for HIV cure-related research at the EOL.

## The Last Gift study

**Fig. 1:**
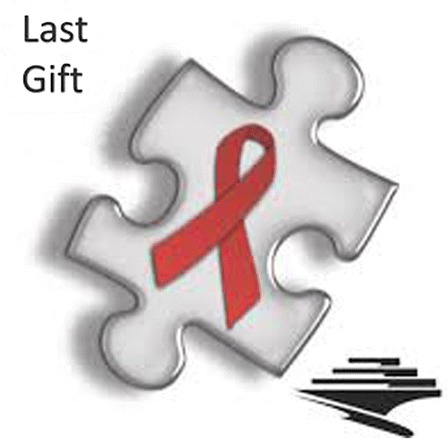
The Last Gift logo was designed by Andy Kaytes, HIV activist and chair of the AntiViral Research Center Community Advisory Board. Each piece of the puzzle represents one Last Gift participant

The Last Gift is a clinical research study at UCSD for PLWHIV who have a terminal illness, such as a solid organ cancer, advanced heart disease, or neurodegenerative disease (e.g., amyotrophic lateral sclerosis (ALS)), and have a prognosis of living less than 6 months, as determined by their physician (Fig. 1) [10]. Approximately 30 Last Gift study participants will be recruited on the basis of their voluntary altruistic motivations to donate their blood and other samples (ante-mortem) and bodies (post-mortem) to advance HIV cure science. Ante-mortem procedures involve basic blood draws and optional collection of genital secretions and rectal swabs to characterize cellular reservoirs of HIV, concurrently with in-depth socio-behavioral sciences questionnaires to assess experiences and feelings about EOL HIV cure-related research participation. Post-mortem procedures include methodical characterization of the size, distribution, activity and mechanisms of HIV reservoirs throughout the body (including blood, brain, genital tract, gut and other deep tissues), based on a rapid research autopsy performed within 6 h of death. If a research autopsy is not performed within 6 h of death, virus and cells degrade and the physiology changes [16]. Answers may inevitably be lost as to why HIV persists in cells and tissues. If Last Gift participants elect to interrupt their HIV treatment before death, the study team will characterize rebounding HIV ribonucleic acid (RNA) populations. In general, HIV cure science can potentially benefit from a peri-mortem human research model by allowing laboratory-based technologies – including genomics, proteomics, and metabolomics – to elucidate how HIV persists in deep tissues with or without ART.

The choice of studying terminally ill volunteers living with HIV was motivated by: 1) the absence of any reasonable expectation of direct clinical benefits in most HIV cure research, 2) the manifest desire in this community to ‘give back’ to the HIV research field [17], 3) limited opportunities for terminally ill PLWHIV to participate in HIV clinical research in general [18], 4) the fact that people at the EOL may be willing to accept higher risks for research participation, 5) the possibility for donating their full body for a rapid research autopsy, and 6) the opportunity to create a new translational research model to advance HIV cure science (e.g. to test novel HIV cure-related interventions in a human model).

Engagement efforts and preliminary acceptability research in Southern California revealed that EOL HIV cure-related research is widely accepted in the local community [19]. This is likely because many older adults living with HIV have survived an epidemic that was once untreatable [3]. Similarly, cancer research has benefited from peri-mortem and rapid autopsy research programs, providing a better understanding of cancer mechanisms and how various oncology drugs have affected these cellular mechanisms [16, 20]. Providing an opportunity for terminally ill persons to make important contributions to science is aligned with the principle of distributive and representational justice [11].

## Ethical principles for clinical research at the EOL

Most HIV cure-related studies consist of observational assessments to better understand HIV reservoirs in the body or proof-of-concept experiments with no expectation of direct clinical benefit [21]. The aims of these studies are to obtain generalizable knowledge and identify novel approaches to cure disease [22, 23]. EOL HIV cure-related research faces many of the same ethical challenges surrounding early-phase research, such as the need to ensure social value and scientific validity of the study, to carefully assess risks, and to ensure voluntary and informed consent [24, 25]. In their seminal article ‘What makes clinical research ethical?’ [26], Emanuel and colleagues outlined seven requirements that provide a strong ethical foundation for clinical research. Their ethical framework was drawn by synthesizing the literature on the ethics of research involving human participants, including the Nuremberg Core (1947), the Declaration of Helsinki (1964, 1975, 1983, 1989, 1996), the Belmont Report (1979) and the International Ethical Guidelines for Biomedical Research Involving Human Subjects (1982, 1993), among others. The seven requirements include: 1) social and scientific value; 2) scientific validity; 3) favorable risk-benefit ratio; 4) informed consent (autonomous choice); 5) respect for participants; 6) fair selection of research participants; 7) independent review. In this paper, we emphasize the first five requirements. In the EOL research context, the principles of social value and scientific progress are paramount, because research participants will have already died by the time scientific findings materialize [27].

Using an approach similar to Emanuel et al., Lo and Grady emphasized eight ethical considerations for the field of HIV cure-related research: 1) collaborative partnership; 2) social value; 3) scientific validity; 4) fair selection of participants; 5) favorable risk-benefit balance; 6) independent review; 7) informed consent; and 8) respect for enrolled participants and communities [13]. Sugarman expanded Lo and Grady’s ethical considerations for HIV cure clinical research to include considerations for third-party risks, confidentiality and media attention, and financial aspects [14]. In this paper, we examine the context of HIV cure clinical research at the EOL and generate considerations adapted to the reality of a specific HIV cure clinical research protocol called *The Last Gift: Development of End-of-Life Translational Research Model.*

### Clinical research at the EOL, rapid research autopsy and gifting relationship

The EOL clinical research literature, particularly in the oncology field, is rich with considerations of ethical conduct of research [11, 12, 27, 28]. Seppet and colleagues, for example, framed key ethical issues for research with dying individuals, including recruitment considerations for terminally ill participants, establishment of inclusion and exclusion criteria, provisions to ascertain sex and gender differences, assessment of capacity to provide informed consent, protection of privacy, and biobanking issues [29]. While the notion of EOL clinical research is not in itself ethically problematic, it can be difficult to conduct such research well given the inherent practical and logistical challenges [30, 31]. For instance, EOL research occurs at the same time as participants and their loved ones prepare for death [3]. More specific to the Last Gift study, one methodological issue that arises is the uncertainty associated with the EOL prognosis [32]. Researchers need to ensure that the timing of research initiation is right – in this case, when life expectancy is 6 months or less. Should participants live longer than 6 months, the research team should be prepared to follow the study participants until the EOL. If that is the case, the Last Gift study procedures do not change, and the research team continues to follow the Institutional Review Board (IRB)-approved protocol. The research team will continue to follow participants as long as needed. If participants are stable for a long time, however, the research team may consider increasing the visit intervals so as to minimize the burden of longitudinal study participation. This should be done on a case-by-case basis. Further, the research team should be careful not to induce death-related distress, meaning that participants would become anxious consenting for a study that requires a prognosis of less than 6 months. This challenge is addressed by carefully selecting participants who are fully aware of their prognosis and have accepted the fact that they are terminally ill.

Another question is the generalizability of EOL scientific data when most HIV cure-related research is intended for ‘otherwise healthy individuals’ [8]. Generalizability of study findings from the Last Gift study to all PLWHIV cannot be ensured due to a small sample size (*n* = 30) and confounding factors associated with the various causes of death. Further, the situation of characterizing the HIV reservoirs of Last Gift participants before and after death is unique to this specific protocol. However, the overall HIV reservoir size and composition should not necessarily be different in PLWHIV at the EOL, particularly in organs that are not affected by the terminal condition. Although some results may not be generalizable to the wider population of PLWHIV, EOL clinical research may be more generalizable to the human condition than non-human animal models, analysis of tissues from repositories without clinical information, or HIV latency in in vitro models [9]. The Last Gift study team attempts to enhance generalizability of ante-mortem data by studying clinical samples collected from otherwise healthy cohorts of individuals who interrupted ART (as part of ongoing or past clinical studies). Research findings from blood donations between the Last Gift and other ART interruption cohort study participants will be compared. If similar patterns in the blood are seen, the team will be more confident in generalizability of findings from the autopsy specimens of the Last Gift cohort. Generalizability will be harder to prove for tissue samples, since most cannot be collected from living individuals.

Another consideration for the case of the Last Gift study is that participants are enrolled in a clinical study to advance HIV cure research, and not to advance understanding of the disease they are dying from. This may present an internal conflict for Last Gift study participants. The research team addresses this concern by being open to co-enrollment in other clinical protocols. For example, one of the Last Gift study participant had a rare ALS-related condition and donated part of his spinal cord to a separate research protocol. Last Gift participants can also co-enroll in the California NeuroAIDS Tissue Network (CNTN) to allow better understanding of mechanisms causing neurological and psychosocial impairments in HIV infection [33]. At this juncture, however, the Last Gift study is purely observational which limits potential study conflicts. Should the Last Gift study include HIV cure investigational interventions (e.g., broadly neutralizing antibodies) that affect clinical research findings in the future, participants may need to choose between enrollment in this HIV cure-related protocol or advancing science for the disease that will cause their death. The specifics would need to be detailed in the inclusion/exclusion criteria of each clinical research protocol.

While ethical frameworks inform clinical research with living human participants, ethical guidelines for research on the recently dead remain limited [34, 35]. The Last Gift study protocol involves a rapid research autopsy that must occur within 6 hours of death. Rapid autopsies are often used in cancer research and allow preservation of proteins and nucleic acids and access to tissues inaccessible during life [16, 20]. This means that autopsy procedures must occur in the emotionally charged environment shortly following the participant’s passing, and the family must be willing to let go of the body [16, 36]. Rapid autopsy protocols need to be robust and study staff must remain continually available. Pentz and colleagues further advised that researchers should treat the body of the newly dead in a dignified manner [35]. For example, Last Gift always observes a minute of silence at the beginning of the autopsy to honor the life of each participant and the gift they gave at death. Invasiveness should also be minimized and justified in terms of expected scientific benefits to be generated [35]. Finally, the ante-mortem wishes of the deceased person should be respected (see Patient-Participant Centeredness discussion below) [37].

Moreover, due to the invasive nature of the study investigating reservoir sites and deep tissues post-mortem, Last Gift study participants must donate their entire body after death. The full body donation should not be confused with traditional organ donation programs [38]. With the passage of the HIV Organ Policy Equity (HOPE) Act, PLWHIV are now able to donate their organs. There is a high (79.8%) willingness in this population to serve as donors [39]. While traditional organ donation is intended for future transplantation into another person, rapid research autopsies use organs and tissues for basic science research [38]. Thus, Last Gift study participants enter into an explicit gifting relationship of their entire body with the study team, other PLWHIV and the HIV cure research community [38, 40]. In the United States, the Uniform Anatomical Gift Act (1968, 2006) governs the making of body donations in biomedical research, and prescribes the conditions by which such gifts can be made [41]. Purposes of body or organ donation can include transplantation, therapy, research or education. The Last Gift study performs a rapid research autopsy and not a clinical forensic autopsy. No individual autopsy report will be shared with family member or loved ones, but scientific results will make sense in the aggregate.

### Protecting autonomy through informed consent

Since there is no clinical benefit of participating in the Last Gift study, a comprehensive informed consent process that distinguishes between benefits to science and benefits to participants is important [42, 43]. The informed consent process must clearly state that EOL HIV cure-related research is not curative – neither for HIV nor the terminal illness – with extreme sensitivity to the language used to describe the research [14, 44, 45]. The informed consent process must also convey the research objectives, methods, procedures, repeatedly emphasize the right to refuse enrollment or withdraw at anytime, and ensure that potential participants understand that their enrollment decision will not affect health care services participants would normally receive [11, 46]. There should also be a formal assessment of the participant’s comprehension about key aspects of the study [13].

In the Last Gift study, the risk of therapeutic misconception – defined as believing incorrectly that the primary purpose of research is to provide clinical benefit rather than advance scientific knowledge [47] – is minimized because participants volunteer for a study distinct from their terminal illness. Researchers clearly state that the Last Gift study has absolutely no curative intent, and research participation is not done out of desperation to become cured of HIV or the terminal illness. Participation in the Last Gift study will not extend life further. Nonetheless, we acknowledge that while therapeutic misconception might not apply to participants at the spoken level, it may exist at the unspoken or implicit level. For example, the need for participants’ social desirability, mediated by altruistic intent, may play an outsized role in their decision to participate. In the Last Gift study, we ensure that volunteers understand they will not benefit clinically from their participation in research, but their donation will help advance the field of HIV cure-related research. Horng and Grady used the term ‘therapeutic optimism’ to describe the hope for best possible outcomes in clinical research [48]. Therapeutic optimism is less problematic because it does not compromise personal autonomy [48]. In the Last Gift study, therapeutic optimism should not be directed towards self given poor prognosis but towards the HIV cure research enterprise as a whole.

Moreover, study candidates should enroll in the Last Gift study with the capacity to consent for themselves. Surrogate consent can be problematic and needs careful consideration [49, 50]. Although current U.S. federal regulations allow for consent to be obtained from a surrogate, this process is also subject to applicable state and local regulations. To date, few states have promulgated specific laws or policies for obtaining surrogate consent for research, particularly research that does not offer the prospect of direct therapeutic benefit to the study participants. Given this unclear regulatory framework, the potential vulnerability of dying patients, the psychological and emotional stress associated with making clinical and research decisions at the EOL, and numerous studies suggesting that surrogates often do not know a patient’s preferences regarding both clinical treatment and study participation, the ethical obligation to protect the rights and wellbeing of study participants suggests that obtaining proxy consent should be carefully evaluated and discussed on a case-by-case basis.

Of related importance, autonomy is determined by three conditions: 1) intentionality, 2) understanding, and 3) a lack of controlling influences [51]. The broader EOL literature shows concerns about decisional capacity of terminally ill persons, due to the possibility of cognitive impairment, which can lead to inability to make informed or rationale choices [46]. Furthermore, HIV-associated cognitive impairments are common among PLWHIV [52]. In the Last Gift study, participants are assessed for cognitive functioning prior to entry to ensure that they are able to understand the study information and make an informed decision.^1^ Anecdotally, participation in the Last Gift study appears to offer participants an opportunity to exert their self-determination and rise above their circumstances despite their imminent death. The Last Gift study team also helps study participants draft their advance directives and a copy of these directives are placed in their study chart. These are important from an ethical standpoint because they respect the autonomy and dignity of the participants.

While informed consent can be seen as a static or one-time event, disease progression is dynamic and may result in diminished capacity over time [11, 53]. The Last Gift study team emphasizes informed consent as a continuous process throughout the entire study and builds the researcher-participant relationship with careful considerations for the participant’s wishes until the EOL. For example, Last Gift participants are asked if they are willing to donate blood until the EOL at each visit. Beaver and colleagues called this approach ‘process consent’, allowing for a renegotiation around participation at different stages of the research interaction [30]. Mackin and colleagues [54] described the process of adaptation as follows:

“For individuals at the end of life, promoting patient self-determination requires a climate in which decision making is both facilitated and adaptive (i.e., accommodating to changes in attitudes on the basis of real-time experiences) at a time when control over various aspects of life is in decline. Vigilance is required to assure patients maintain their rights of self-determination for as long as possible, only relinquishing control to a proxy who has a deep appreciation of the individual’s values and desires. This is *best accomplished far in advance of serious declines in personal health*, and, practically speaking, prior to discussions concerning participation in a specific research protocol.” [*Emphasis added*]

Additionally, considerations should be given to the EOL stages in the context of research, such as the transition from primary medical care, to palliative care on to hospice care, to the dying process, and finally to the impact of death on the loved ones and next-of-kin (bereavement process) [3].

An important aspect of informed consent is ensuring that the process remains free from coercion or undue influence [11]. Agrawal defined coercion as “[a] credible and irresistible force exerted by one person that negatively limits the options of another person” [12]. Coercion is thus a relational concept that undermines voluntariness [12]. For example, the influence of the physician, biomedical HIV cure research team, or family members could create pressure to enroll in a clinical study [12]. Labeling terminally ill patients at the EOL as inherently coerced, however, may fail to take into consideration the various pressures that may exist at the EOL. In the Last Gift study, participants do not enroll out of desperation to be cured of HIV as most of them have done well on antiretroviral therapy. Possible ways to reduce coercion would be to ensure that the physician taking care of patients at the EOL would not be the one obtaining informed consent from candidates to enroll in clinical research, so that the demarcation between clinical care and research participation remains clear [12, 54]. Similarly, as part of the Last Gift study, researchers must be careful not to interfere with the clinical care team by giving medical advice to study participants. Of note, the Last Gift researchers and clinicians remain separate, and each Last Gift participant has his/her own clinical team. The Last Gift team does not provide clinical care, not even palliative care. The Last Gift team also does not interfere with any medical decision made by the participant’s clinical care team.

Another method considered by Casarett involves giving candidates ample time to make decisions over multiple visits [46]. The informed consent process and research interactions must emphasize the voluntary nature of participation that can be revoked at anytime and for any reason without consequences [54]. Further, financial incentives should not be the primary motivator for participation in the Last Gift study. The Last Gift study provides modest compensation for blood draws and supports costs for cremation post-research autopsy if the participant desires. After extensive discussion with the Community Advisory Boards and the UCSD IRB, it was deemed that cremation would not exert an undue inducement in the Last Gift study, but was a necessity given the invasive nature of research procedures post-mortem. Cremation, however, is not described as a study benefit.

The Last Gift study team favors shared decision making as a way to enhance the recruitment experience for study participants. Decisions to enter in the study are understood as a set of interactions with the research team, rather than discrete, isolated events [55–58]. The research team helps Last Gift candidates understand that a decision should be made, while describing the risks, benefits, uncertainties and possible options to candidates, to ultimately help them come to a decision [57]. Shared decision making also helps promote trust between the research team and study participants [56]. As explained by Epstein and colleagues:

“Engaging patients in constructing preferences in the face of complexity, inadequate evidence, and irreducible uncertainty involves more than provision of information and an invitation to choice. It also involves dialogue about the communication process itself; that is, what patients want to know, what information is relevant, how patients prefer to be informed, patient’s roles in decision making, and who else (if anyone) should be present. Seen in this way, constructing preferences (…) involves building relationships, providing information, and exploring preferences, which then strengthen relationships, understanding, and involvement in decisions” [55].

In such highly innovative HIV cure-related research, investigators must interact with study participants as true partners and collaborators in research. In the Last Gift, participants help investigators answer scientific questions [43]. Study participants must be able to make decisions that are free of coercion, manipulation or undue persuasion [49].

### Avoiding exploitation and fostering altruism

Agrawal defined exploitation as “[t]he unfair distribution of the benefits and burdens from a transaction” [12]. Protection from exploitation in clinical research is largely related to the ethical principle of distributive justice, in that the individuals or community taking on the burden of research derive some sort of compensatory benefit. Since EOL HIV cure-related research does not confer direct clinical benefit, a worry arises that terminally ill PLWHIV are inherently subject to exploitation [59]. If participants genuinely share the goal for which research is conducted, as is believed to be the case of the Last Gift study, the exploitative worry is diminished [59]:

“If research subjects have genuinely altruistic motives, that fact can make a direct difference to the ethical permissibility of a clinical trial: it can remove concerns about exploitation that would otherwise apply.”

We believe that altruism can remove some concerns about exploitation that would otherwise apply. As such, we believe that both avoiding exploitation and fostering altruism should receive attention in EOL HIV cure-related research. Hence it is worth exploring the concept of altruism in the context of the Last Gift study. Lee defined altruism as “unselfish concern for the welfare of others [60].” Research shows that altruism is not a sole factor in a person’s decision-making process, but it is nevertheless important [61]. The literature suggests that people rarely participate in clinical research out of purely altruistic reasons. They also hope to personally benefit from the transaction [61]. Bidad and colleagues made the distinction between pure altruism (true selflessness), hypothetical altruism (stated selfless behavior but not put to the test), weak altruism (hope to personally benefit), contingent altruism (altruistic behavior contingent upon personal benefits) and sense of duty [61]. While these different types of altruism have oftentimes been cited reasons to participate in HIV cure clinical research [62–65], altruism has not been well described in the HIV cure research context. We suggest the need for further empirical research examining the psychological characteristics of Last Gift study participants to understand why they decide to participate in EOL HIV cure-related research and take on additional risks and burdens for the benefit of others [66]. In particular, altruism should be explored as the construct of weighing societal benefits of research participation above personal risks and burdens [67]. Understanding altruistic motivations of PLWHIV at the EOL could help inform recruitment, informed consent, retention and overall study implementation [38]. Undoubtedly PLWHIV should be permitted to assume risks to help advance HIV cure science. While the presence of altruistic motivations may reduce concerns of exploitation, there should nonetheless be limits placed onto altruism [68]. Różyńska justified the imposition of limits on risks in clinical research by the need to protect both the research enterprise and the study participants [69]. These limits are usually determined by the IRB and regulatory bodies overseeing clinical research [12]. The study team also has an ethical responsibility to question why Last Gift participants decide to join the study, and ensure that they do so out of altruism and for the societal and scientific benefits [38].

Furthermore, even when the nature of a transaction seems unfair, as in the case of EOL HIV cure-related research, there is nothing unethical or exploitative in pursuing one’s self-interests. This is particularly true when the goals are reasonable and altruistic, as is true in this case. EOL HIV cure-related research has, prima facie, the goal of improving the lives of future PLWHIV. Moreover, there is a direct but immeasurable benefit to participants through their altruistic contributions. Wertheimer acknowledged this when he wrote that “we need a more protean conception of what counts as a benefit to A, one that includes A’s purposes, goals and values [70].” Other philosophers have made similar arguments, such as Feinberg’s proposition that the notion of benefit should include “fulfillment of one’s aims, purposes, or desires, including altruistic and conscientious ones [71].”

### Maintaining a favorable benefits to risks balance

In accordance with the principles of beneficence and non-maleficence [49], research must be carefully assessed to ensure that risks remain balanced in relation to benefits for participants and society [26, 72]. Clinical research should minimize risks, enhance potential benefits, and ensure that risks and burdens are justified in relation to prospective benefits [26]. Benefit-risk assessments can be challenging, however, because they are contextual and asymmetric (e.g. risks are often to participants while benefits are to society) [8]. Another challenge is that benefits and risks may differ from the perspectives of the IRB versus the participants [8]. Participants may see payments, services or altruism as benefits, whereas researchers and IRB members are prohibited from considering them thus. Further, risks can be relative to a consenting individual’s current medical condition, like the stage of disease and care options available [8]. Weijer and Miller called for the use of a ‘risk-knowledge calculus’ to determine whether risks of clinical research could be justified [73]. In the Last Gift study, we must consider what constitutes favorable benefit-risk ratios for both HIV cure-related research *and* PLWHIV at the EOL. Participants have no survival expectations beyond 6 months. Moreover, research information is not designed to alter disease course or prolong survival, but contribute to generalizable knowledge about HIV reservoir research [11]. This contrasts to other EOL research whereby terminally ill patients may be willing to undergo aggressive and risky investigations with the hope of prolonging life.

Benefits, risks and burdens of research at the EOL may also be difficult to define and change over time. For example, Casarett and Karlawish described participant goals at the EOL in terms of relief from symptoms, dignity and meaning, social relationships and control [46]. Time spent undergoing blood draws and answering questionnaires may detract from valuable time with loved ones [11]. While scientific review committees usually consider risks in clinical terms, it is important to pay attention to psychosocial aspects of clinical research participation at the EOL [11]. Terminally ill persons may derive tremendous mental and emotional benefits from research participation, especially when able to make a lasting contribution to science [54, 74]. A critical synthesis of the EOL clinical research literature revealed that most terminally ill clinical study participants viewed the experience as positive, but a minority experienced minor psychological distress [27]. Careful selection of EOL study participants and proactive identification of concerns can help minimize the possibility of distress [16]. While our preliminary experience with the Last Gift study showed that research participation has been overwhelmingly positive for participants, more empirical research is needed to determine how terminally ill PLWHIV experience HIV cure-related research participation at the EOL in order to guide investigations and ethics reviews. Such research will be critical if research teams hope to maintain a favorable balance of benefits to risks that terminally ill PLWHIV will also find acceptable [46]. Further, PLWHIV, other terminally ill individuals, and families/loved ones of recently deceased patients should be included in EOL HIV cure research design and IRB deliberations to help define acceptable benefits and risks in such research.

One ethical question that arises in the context of the Last Gift study is whether PLWHIV would be willing to accept greater risks in order to participate in interventional EOL HIV cure clinical research. While the current Last Gift study is limited to observational assessments (e.g. what happens to viral reservoirs when ART is maintained or interrupted), PLWHIV may be willing to take part in experimental research involving immune-based strategies, latency-reversing agents, or other HIV cure research approaches tested in non-EOL volunteers. Involving terminally ill PLWHIV in interventional HIV cure research should be considered since first generation research will likely involve significant toxicities and burdens. Like first generation antiretroviral therapies, their effectiveness will likely be suboptimal or even harmful.

We propose a benefit-risk assessment framework similar to the one suggested by DiGiusto and colleagues [75], which compares the ethical permissibility of testing HIV cure-related research interventions with PLWHIV at the EOL (Table 1). Such assessments must take into consideration the population under study, the risks of the interventions and other clinical factors [8, 76]. We believe that latency-reversing agents and immune-based strategies would have a favorable benefit-risk profile for PLWHIV at the EOL, while stem cell transplantations would be unfavorable. An important consideration is that some interventions are too risky and not adequate to be offered to terminally ill participants (e.g. stem cell transplants requiring total ablative chemotherapy and radiation). Further, long-term safety and effects would not become manifest with terminally ill participants because of the short expected life-span. The EOL translational model, however, might still be useful to evaluate some parts of an intervention. For example, in the case of gene editing, while long-term follow-up of study participants would be needed to test for potential genotoxic effects, the EOL translational research model could still be useful to determine how editing techniques can safely be delivered to cells and tissues – including the brain. Additionally, we believe analytical treatment interruptions would have a favorable benefit-risk ratio at the EOL and are already occurring as part of the Last Gift study. For example, many PLWHIV at the EOL no longer wish to continue their ART. For observational studies, however, terminally ill PLWHIV should not be explicitly asked to interrupt ART, but should elect to do so on their own. Some interventional studies – such as those involving immune-based strategies – may include an analytical treatment interruption in the study design. Finally, the physical state of the participant should also be taken into consideration when undergoing complex blood draws (e.g., leukaphereses) or other invasive study procedures (e.g. lumbar punctures and biopsies), and therefore, case-by-case determinations should be made.

**Table 1:** Benefit-Risk Assessment for HIV Cure-Related Research at the EOL

HIV Cure-Related Research Approaches	Possible Positive Outcomes	Potential Risks	Benefit-Risk Assessment for PLWHIV at the EOL	Other Considerations
Latency-reversing agents	Stimulation of replication-competent provirus from latently-infected cells and perturbation of HIV reservoir (although this has no direct clinical benefit)	Side effects of latency-reversing compounds (various toxicity levels)	Favorable	Already being tested in ‘otherwise healthy volunteers’; long-term effects would not become manifest
Immune-based strategies (e.g. broadly neutralizing antibodies)	Improved immune response to HIV	Risks of immune-based approaches, including potential for development of auto-immunity	Favorable	Already being tested in ‘otherwise healthy volunteers’; long-term effects would not become manifest
Stem cell transplants	Modality aims at making cells resistant to HIV infection	Risks associated with irradiation and chemotherapy; hepatic effects, renal failure, graft-versus-host-disease (GVHD); too great to withstand for PLWHIV at the EOL	Unfavorable; cannot be justified in PLWHIV at the EOL	Not indicated in ‘otherwise healthy volunteers’; engraftment and chimerism may not have time to manifest at the EOL
Gene editing or modification	Process of editing deoxyribonucleic acid (DNA) inside immune cells to make them less susceptible to HIV or better able to clear infected cells	Off-target modifications or activation of proto-oncogenes causing malignancies; other risks associated with gene editing	Contingent on the intervention	Long-term effects would not become manifest; EOL translational research model could still be useful to determine how gene editing techniques can be delivered safely to cells and tissues – including the brain
Analytical treatment interruptions	N/A	Development of resistance to ART regimen; increased risks of developing opportunistic infections; other risks associated with analytical treatment interruptions	Favorable	Already being conducted in Last Gift study – in observational studies, PLWHIV should not be explicitly asked to interrupt ART, but should elect to do so on their own; some interventional studies (e.g. immune-based strategies) may include an analytical treatment interruption in the design

### Safeguarding against vulnerability through patient-participant centeredness

The unique circumstances surrounding the EOL period may imply that some participants are potentially vulnerable [11]. Vulnerability refers to the “[i]ncreased potential that one’s interests cannot be protected [12].” In clinical research, the epithet ‘vulnerable’ denotes specific requirements for protecting or safeguarding participants’ safety and rights. The U.S. Federal regulations for the protection of human research participants classify children, pregnant women/fetuses/neonates, prisoners and persons with mental disabilities as vulnerable [77]. The NIH has also adopted a broader definition of vulnerability in which terminally ill individuals are also considered a “special class of research subjects [78].” Interestingly, research with deceased humans does not fall under the technical definition of research with human participants [34, 77] and the U.S. Office for Human Research Protections (OHRP) remains conflicted whether research with the newly dead meets the technical definition of human research [79].

A number of authors view people at the EOL as inherently vulnerable due to their terminal condition [30, 80, 81]. Other scholars prefer to explicitly define what people are vulnerable to for the designation to hold salience in the clinical research context [12]. For instance, Kipnis identified six causes of vulnerability that might interfere with a participant’s ability to provide informed consent: 1) cognitive, 2) deferential, 3) medical, 4) juridic, 5), allocational, and 6) infrastructural vulnerability [82]. Henry and Scales posed that terminally ill individuals should be considered vulnerable if they lack decision-making capacity from cognitive impairment due to an underlying disease, complication or side-effect of therapy [11]. Moreover, dying individuals can be vulnerable if their medical state makes them susceptible to therapeutic misconception out of desperation, as discussed above [11]. Agrawal recommended that vulnerability be evaluated on a case-by-case basis, and concluded that it does not necessarily lead to lack of voluntariness [12].

We do not believe Last Gift study participants should be categorized as vulnerable simply because they are terminally ill. Instead, the potential for vulnerability should be carefully evaluated in the clinical research context for each participant [11, 83, 84]. Further, asking about specific aspects of a person or the circumstances that might render participants vulnerable serves as a basis for ethical and effective clinical study implementation [85]. Pre-empting possible causes of vulnerability can lead to improved protections while avoiding unnecessary barriers to participation, stereotyping and even stigmatization [84]. In fact, older PLWHIV who were infected decades ago when HIV was a death sentence have faced their own imminent mortality once before and are arguably more capable of making decisions to enroll in HIV clinical research. To deny them the opportunity to contribute to HIV cure-related research at the EOL based on an a priori label of vulnerability would seem to be overly paternalistic [27]. The HIV community dealt with similar issues in the 1980s, when sick participants who did not meet inclusion/exclusion criteria were excluded from early, life-saving HIV treatment trials. PLWHIV fought for compassionate use and expanded treatment access, which provided valuable data in the clinical setting where emerging antiretrovirals were eventually used.

Moreover, EOL clinical research requires careful design and execution, as well as sensitivity to participants’ needs [27]. Terminally ill individuals have specific physical, psychological, social and spiritual needs while preparing for death and achieving life closure [74, 86]. We believe in the need for patient-participant-centered clinical research with terminally ill PLWHIV. In a study of patient needs and preferences at EOL, twenty-six (26) items were considered consistently important, including pain and symptom management, preparation for death, achieving a sense of completion, decisions about treatment preferences, and being treated as a whole person [74]. In a different review, fatigue was the most common and distressing symptom associated with HIV, affecting 20 to 60% PLWHIV in the general population [3]. Pain is also a symptom that has received attention in research with PLWHIV [87]. A systematic review of 61 studies found that the prevalence of pain (defined operationally as a symptom) among PLWHIV ranges from 54 to 83% [88]. Fatigue and pain are likely to be exacerbated when coupled with a terminal illness. In addition to fatigue and pain, mental health burdens [89], social isolation [90, 91], insomnia [92] and body image issues [93, 94] may be of great import to PLWHIV. Depression, in particular, is of clinical relevance and may need careful evaluation and treatment, beyond the provision of psychosocial support [95, 96]. Further, our experience reveals that some interviews with terminally ill PLWHIV require frequent interruptions, with hospice nurses coming in and out, and participants asking for various items. Study staff must remain extremely flexible when conducting HIV cure-related research with PLWHIV at the EOL.

An issue that has emerged in the Last Gift study is the necessity to maintain boundaries between the Last Gift team and the study participants, and to maintain good communication with clinical teams and providers. The Last Gift team spends a lot of time with study participants and often create special bonds with each of them. As a consequence, Last Gift participants trust the research team and discuss very intimate thoughts, concerns and wishes [97]. The Last Gift team observes participant centeredness by bringing favorite foods and providing other small attentions to study participants. This shows the appreciation, reverence, gratitude and respect the research team has for the participants. None of these niceties are presented as benefits of the study to the participants but are considered small tokens of appreciation for their altruism and acknowledgements of scientific contributions.

Another point of considerations is the need to not be overbearing with study procedures, especially at the very end of life. For example, the Last Gift protocol indicates blood collections as close to death as possible. We specifically ask study participants early on if they are willing to donate blood until the very EOL, including when they will be sedated or on life support. We must also ensure that the next-of-kin/loved ones feel comfortable honoring the Last Gift participants’ wishes until the EOL. Our experience has shown that it takes a very close relationship and sensitive approach to accomplish this without seeming too intrusive. Further, drawing blood at the EOL may present challenges, since terminally ill people may be dehydrated, with low blood pressure, or fragile veins.

Table 2 describes possible examples of patient-participant-centeredness considerations for the Last Gift study (note: this list is not exhaustive).

**Table 2:** Examples of Patient-Participant Centeredness Considerations for Last Gift Study

EOL Clinical Research Conduct	
• Minimize burden of study participation for terminally ill participants [54]	
• Ensure research remains flexible, taking into consideration fatigue and fluctuating symptoms across disease trajectory [27, 54]	
• Assist participants with completion of study procedures and questionnaires [27]	
Quality of Life at the EOL	
• Pay attention to quality of life at the EOL [12]. For example, location of care is an important indicator of quality of EOL care [109].	
• Honor treatment preferences of terminally ill individuals, including pain management and palliative care [110]	
• Respect participants’ privacy and need for time with next-of-kin/loved ones	
• Consider participants’ food preferences and other small attentions	
• Consider how substance use at the EOL affects study participation (e.g. alcohol, cannabis)	
Advance Care Planning	
• Assist participants with advance care planning needs [74]	
• Provide proper referral and counseling for participants who desire medical aid to end life under California End of Life Option Act (EOLOA) of 2016	
Mental Health, Cultural and Spiritual Issues	
• Provide adequate psychosocial support to study participants. The Last Gift study team has a two psychiatrists and one licensed psychologist on staff.	
• Give consideration to mental health issues of participants, including fear, suicide ideation, depression, among others [28]	
• Pay attention to cultural issues, spiritual well-being and meaning as integral to the dying process [3, 28]	
Financial and Legal Issues	
• Pay attention to issues around the burden of cost of dying and health insurance	
• Help ensure participants have support for EOL legal needs [111]	

The California End of Life Option Act (EOLOA) went into effect in June 2016, creating a process for dying patients to ask doctors for a prescription for medical aid-in-dying that patients can ingest privately at home [98]. Like laws in six other U.S. states and the District of Columbia, the California EOLOA gave mentally capable, terminally ill adults with 6 months of less to live the option to request medications to end unbearable suffering and die peacefully. Although the future of this law is uncertain [99, 100] and it is beyond the scope of this paper to discuss the ethics of medical aid to end life, we acknowledge ethical tensions related to individual autonomy versus paternalism, the nature of the patient-physician relationship, trust in that relationship and the medical profession, and the role of the medical profession in society [101]. In a study of 70 patients receiving palliative care for advanced cancer, 73% believed that medical aid to end life should be legalized, 58% said they would consider it if symptoms became insufferable, and 12% would have made the request at the time of the interview [102]. The main reason for favorably viewing medical aid to end life was the relief provided by a straightforward death [102]. The End of Life Option Act creates an ethical tension for Last Gift study participants who desire such prescription, as the decision to die must not be done autonomously and independently but must also not be coerced or made under undue influence. The study team should also be prepared to properly consult and refer Last Gift participants if they ask for such provision. Given the nature of the rapid research autopsy, participants’ intentions should also be made known to the next-of-kin/loved ones and/or the study team.

### Ensuring acceptance of next-of-kin/loved ones and community

In addition to safeguarding patient-participant centeredness, it is important to ensure acceptability of the research from next-of-kin/loved ones and the broader community. By next-of-kin/loved ones, we mean significant others, family members, friends and close acquaintances of Last Gift study participants. By community, we mean “a group of people with diverse characteristics who are linked by social ties, share common perspectives, and engage in joint action in geographical locations or settings” [103], such as the broader community of PLWHIV.

As our study team experienced, next-of-kin/loved ones often have a significant role to play in decision-making and research participation [17]. We believe next-of-kin/loved ones should be informed of the participants’ wishes and we should clarify their role in the study [16, 104–107]. For example, our research plan clearly states that consent for blood draws and rapid research autopsy will be discussed with a next-of-kin/loved ones in order to minimize concerns or conflicts during the research and at the time of death. Optimally, the research context should go beyond minimizing concerns and conflicts with the research, to also actively providing support and protecting next-of-kin/loved ones. We also acknowledge that the notion of next-of-kin/loved ones may include untraditional arrangements for PLWHIV.

Further, our study includes a socio-behavioral sciences component with next-of-kin/loved ones of Last Gift participants to assess perceptions and attitudes towards the study. This activity occurs twice during the study: 1) shortly after the Last Gift study participants enroll in the study, and 2) shortly following death. This provides an opportunity to talk about loss and the bereavement process, meaning of study participation, any logistical or practical issue encountered and ways to improve the conduct of the study. The Last Gift study team takes care of logistical and administrative items (e.g., transportation, vital statistic worksheet, death certificates) to take stress away from next-of-kin/loved ones during difficult times. We also provide appreciation letters to next-of-kin/loved ones after the study has ended. The Last Gift team also tries to follow-up with the next-of-kin/loved ones after death to make sure they are recovering and have adequate social support.

Finally, we must be sensitive to socio-cultural issues and ensure community acceptance around the study. Following Good Participatory Practice [108], our team conducted extensive consultation sessions with the UCSD AntiViral Research Center Community Advisory Board, the Palm Springs HIV and Aging Research Project and the Palm Springs Positive Life Program prior to study initiation. There was unanimous approval of the project by various groups and an overall community willingness to support the study. Such community interactions will be ongoing and eventually involve data dissemination. We will focus on describing how participation in research contributes to advancing HIV cure science.

## Conclusions

In sum, we examined considerations for the ethical conduct of EOL HIV cure-related research. The Last Gift study at UCSD enrolls terminally ill PLWHIV who have a prognosis of less than 6 months and desire to contribute to HIV cure-related research. Ethical considerations spanned five domains: 1) protecting autonomy through informed consent, 2) avoiding exploitation and fostering altruism; 3) maintaining a favorable benefits/risks balance, 4) safeguarding against vulnerability through patient-participant centeredness, and 5) ensuring acceptance of next-of-kin/loved ones and community stakeholders. Our considerations are summarized in Table 3; issues described are not comprehensive. Further exploration is needed regarding what the fair selection of participants and independent review would mean for HIV cure-related research towards the EOL. We also strongly advocate for the critical need to include bioethicists and social-behavioral scientists on biomedical HIV cure research teams.

**Table 3:** Ethical Considerations for HIV Cure-Related Research at the End of Life

Themes	Considerations
EOL clinical research, rapid research autopsy and gifting relationship	The research team should: • Anticipate issue of prognostic uncertainty, including ensuring that timing of research initiation is right and be prepared in case participants live longer than 6 months. • Maximize generalizability of EOL scientific data to HIV cure-related research field. • Allow co-enrollment in another research protocol to help advance science for disease participants are dying of, provided there is no inclusion/exclusion criteria conflict. • Follow established guidelines related to rapid research autopsy, including: having staff available at all times, treating the body of the deceased in a dignified manner, minimizing and justifying degree of invasiveness in relation to the expected scientific benefits, and respecting pre-mortem wishes of Last Gift study participants.
Protecting autonomy through informed consent	• The informed consent process must clearly state that EOL HIV cure-related research will not be curative, and must convey the research objectives, methods, procedures, decision will not affect medical care, and right to refuse enrollment or withdraw from research at anytime. • Last Gift study participants should consent for themselves and participants should be assessed for cognitive functioning. • Last Gift study team should view the informed consent process as continuous and carefully consider Last Gift participants’ wishes until the EOL (e.g., process consent) or advance directives. • The informed consent process must remain free from coercion, or undue influence or persuasion. Possible ways to reduce coercion include: 1) physician taking care of patient should not provide consent to enroll in Last Gift study, 2) decisions should be made over multiple visits; 3) research team should emphasize voluntary nature of research; 4) financial incentives should not be primary motivator to participate. • Shared decision making should be used as a way to enhance the recruitment experience, promote trust and view participants as true partners in research. • The risk of therapeutic misconception should be minimized. • For additional considerations, see [12].
Avoiding exploitation and fostering altruism	• Protection from exploitation relates to the principle of distributive justice, in that there should be fair distribution of the benefits and burdens from the transaction. • The Last Gift study team should ensure that participants have genuinely altruistic motives for participation and understand that they are entering a gifting relationship. • Further empirical research is needed to examine the role of altruism and psychological characteristics of Last Gift study participants to understand their decisions to participate in EOL HIV cure-related research. • IRBs and regulatory bodies should place limits on ethically permissible risks of clinical research to protect the HIV cure-related research enterprise.
Maintaining a favorable benefits to risks balance	• EOL HIV cure research should minimize risks, enhance potential benefits, and ensure that risks and burdens are justified in relation to prospective benefits. • A favorable risk-knowledge balance should be obtained, since EOL HIV cure research will not alter disease course or prolong survival. • The research team should pay attention to evolving participant goals, including relief from symptoms, dignity and meaning, social relationships, and other possible psychosocial risks and benefits. • More empirical research is needed to determine how terminally ill PLWHIV view acceptable benefits and risks. • PLWHIV as well as terminally ill individuals and families/loved ones of recently deceased patients should be included in protocol design and during ethics reviews. • Benefit-risk assessments should take into considerations the risks of HIV cure research interventions and other clinical factors. In general, latency-reversing agents and immune-based strategies have a favorable benefit-risk balance, while stem cell transplantation do not. The EOL translational model could be helpful in evaluating components of gene modification and editing (e.g. safe delivery to cells and tissues – including the brain). • Analytical treatment interruptions can be ethically permissible at the EOL; however, participants should elect to interrupt beneficial ART on their own for observational studies. Some interventional study designs may warrant analytical treatment interruptions.
Safeguarding against vulnerability through patient-participant centeredness	• Vulnerability implies the need for specific protection or safeguards. • Last Gift participants should not be categorized as vulnerable simply because they are terminally ill. Rather, attention should be paid to what participants may be vulnerable to, and adequate protections should be put in place to alleviate possible sources of vulnerabilities. • Research with terminally ill PLWHIV requires careful design and execution, and incorporate sensitivity to participants’ needs, including physical, psychological, mental, spiritual and social needs (see Table 2). For example, researchers should pay attention to issues related to depression, social isolation, insomnia, and body image that may be of import to PLWHIV. • Appropriate boundaries should be maintained between the Last Gift team and the study participants. Special attentions such as special foods should not be presented as study benefits, but as tokens of appreciation, reverence, gratitude and respect. • The research team should be careful to not be intrusive while attempting to honor the participants’ wishes until the very EOL.
Ensuring acceptance of loved ones and community	• The research team should ensure acceptability of the research from next-of-kin/loved ones, and clarify the role of next-of-kin/loved ones in study participation. Next-of-kin/loved ones should also be briefed on the study to be willing to let go of the Last Gift participants’ body at the time of death. The research team should actively support and (if needed) protect next-of-kin/loved ones, and account for the fact that next-of-kin/loved ones arrangements may be untraditional for PLWHIV. • The research team should be sensitive to socio-cultural issues. Extensive and ongoing community consultation should take place before, during and after the study. • More empirical research is needed to better understand the perceptions of next-of-kin/loved ones and community stakeholders around HIV cure-related research at the EOL.

Clinical research is essential to advancing science and medical care. Terminally ill PLWHIV should not be denied the opportunity to participate in important HIV cure clinical research opportunities. Once faced with imminent death when HIV was untreatable, several PLWHIV now desire to give back to the field of HIV research. They find a deep sense of purpose and tremendous meaning in death and in leaving a legacy. EOL HIV cure-related research can be done ethically and effectively by proactively anticipating key issues that may arise. While not unique to the fields of EOL or HIV cure research, considerations highlighted can help us support a new research approach. We must honor the lives of PLWHIV whose involvement in research can provide the knowledge needed to achieve the dream of making HIV infection curable.

## Abbreviations

AIDS: Acquired Immunodeficiency Syndrome
ALS: Amyotrophic Lateral Sclerosis
ART: Antiretroviral Therapy
AVRC: AntiViral Research Center
CNTN: California NeuroAIDS Tissue Network
DNA: Deoxyribonucleic Acid
EOL: End of Life
EOLOA: End of Life Option Act
FDA: Food and Drug Administration
GVHD: Graft Versus Host Disease
H+ARP: HIV and Aging Research Project – Palm Springs, California
HIV: Human Immunodeficiency Virus
HOPE Act: HIV Organ Policy Equity Act
IRB: Institutional Review Board
NIAID: National Institute of Allergies and Infectious Diseases
NIMH: National Institute of Mental Health
OHRP: Office for Human Research Protections
PHLP: Public Health Leadership Program
PLWHIV: Persons/People Living with HIV
RNA: Ribonucleic acid
UCSD: University of California San Diego
UNC: University of North Carolina

## Footnotes

1 The Last Gift study team follows the guidelines established by the *Task Force for Recommending Procedures for Determination of Decisional Capacity in Subjects Participating in Research Protocols* that included the following members: Dilip Jeste, MD (Chair), David Braff, MD, Daniel Masys, MD, Barton Palmer, PhD, Martin Paulus, MD, Lucille Pearson, and Larry Schneiderman, MD. This Task Force was convened by Lew Judd, MD, Chairman of UCSD’s Department of Psychiatry at the request of the UCSD Human Research Protections Program.
